# An Innovative Artificial Intelligence Classification Model for Non-Ischemic Cardiomyopathy Utilizing Cardiac Biomechanics Derived from Magnetic Resonance Imaging

**DOI:** 10.3390/bioengineering12060670

**Published:** 2025-06-19

**Authors:** Liqiang Fu, Peifang Zhang, Liuquan Cheng, Peng Zhi, Jiayu Xu, Xiaolei Liu, Yang Zhang, Ziwen Xu, Kunlun He

**Affiliations:** 1 Chinese PLA Medical School, Beijing 100853, China; heartbeats2017@126.com (L.F.); shengxinzhipeng@163.com (P.Z.); jiayxu301@163.com (J.X.); yangzhang@126.com (Y.Z.); xuziwen98@163.com (Z.X.); 2 Medical Innovation Research Department, Chinese PLA General Hospital, Beijing 100853, China; 13521751769@163.com; 3 Key Laboratory for Research and Evaluation of Artificial Intelligence Medical Devices, Chinese PLA General Hospital, Beijing 100853, China; 4 Medical Engineering Laboratory, Chinese PLA General Hospital, Beijing 100853, China; 5 Key Laboratory of Biomedical Engineering and Translational Medicine, Ministry of Industry and Information Technology, Chinese PLA General Hospital, Beijing 100853, China; 6 BioMind Technology Inc., Beijing 101318, China; peifang.zhang@biomind.ai; 7 Department of Radiology, The Sixth Medical Center, Chinese PLA General Hospital, Beijing 100048, China; cheng_liuquan@139.com

**Keywords:** non-ischemic cardiomyopathy, cardiac MRI, deep learning, intraventricular pressure gradient, convolutional neural network, generative adversarial network, biomechanical analysis

## Abstract

Significant challenges persist in diagnosing non-ischemic cardiomyopathies (NICMs) owing to early morphological overlap and subtle functional changes. While cardiac magnetic resonance (CMR) offers gold-standard structural assessment, current morphology-based AI models frequently overlook key biomechanical dysfunctions like diastolic/systolic abnormalities. To address this, we propose a dual-path hybrid deep learning framework based on CNN-LSTM and MLP, integrating anatomical features from cine CMR with biomechanical markers derived from intraventricular pressure gradients (IVPGs), significantly enhancing NICM subtype classification by capturing subtle biomechanical dysfunctions overlooked by traditional morphological models. Our dual-path architecture combines a CNN-LSTM encoder for cine CMR analysis and an MLP encoder for IVPG time-series data, followed by feature fusion and dense classification layers. Trained on a multicenter dataset of 1196 patients and externally validated on 137 patients from a distinct institution, the model achieved a superior performance (internal AUC: 0.974; external AUC: 0.962), outperforming ResNet50, VGG16, and radiomics-based SVM. Ablation studies confirmed IVPGs’ significant contribution, while gradient saliency and gradient-weighted class activation mapping (Grad-CAM) visualizations proved the model pays attention to physiologically relevant cardiac regions and phases. The framework maintained robust generalizability across imaging protocols and institutions with minimal performance degradation. By synergizing biomechanical insights with deep learning, our approach offers an interpretable, data-efficient solution for early NICM detection and subtype differentiation, holding strong translational potential for clinical practice.

## 1. Introduction

Non-ischemic cardiomyopathies (NICMs) represent a heterogeneous group of myocardial diseases characterized by structural and functional abnormalities of the heart muscle, independent of coronary artery obstruction or ischemic injury. This group encompasses diverse conditions such as dilated cardiomyopathy (DCM), hypertrophic cardiomyopathy (HCM), hypertensive cardiomyopathy (HTCM), and cardiac amyloidosis (CA). Accurate and early subtype classification of NICMs is paramount, as these conditions are significant contributors to heart failure, life-threatening arrhythmias, and sudden cardiac death [1]. Furthermore, the inherent heterogeneity in electrophysiological properties at both cellular and tissue levels underscores the critical need for precise diagnostic stratification to guide appropriate management strategies [2].

Cardiac magnetic resonance (CMR) imaging has emerged as the non-invasive gold standard for assessing myocardial anatomy, function, and tissue characteristics, owing to its exceptional spatial and temporal resolution [3]. However, conventional CMR-based diagnosis largely relies on visual interpretation, qualitative scoring, and manually derived volumetric parameters. These traditional methods are often limited by significant interobserver variability and possess insufficient sensitivity to detect subtle or early-stage myocardial dysfunction [4]. Critically, they frequently fail to capture underlying biomechanical dynamics, such as the left ventricular intraventricular pressure gradient (LV-IVPG), which plays a fundamental role in both diastolic and systolic function and may offer deeper insights into the pathophysiology of NICMs [5].

Recent advances in artificial intelligence (AI), particularly deep learning algorithms, offer powerful tools for automated feature extraction and disease classification from complex medical imaging data, including CMR [6]. Convolutional neural networks (CNNs) and other architectures, often enhanced by transfer learning, have demonstrated a remarkable performance in various diagnostic applications [7]. Despite these successes, existing AI models applied to cardiomyopathy classification predominantly rely on anatomical or morphological features derived from standard CMR sequences. Consequently, they often overlook the crucial underlying physiological and biomechanical determinants that drive disease heterogeneity and progression [8].

To address this critical gap, we hypothesize that integrating biomechanical markers, specifically LV-IVPG derived from CMR, with advanced deep learning architectures can significantly enhance the diagnostic and prognostic assessment of NICMs. This study aims to develop and evaluate a novel, integrated framework that synergistically combines the power of deep learning for automated image feature extraction with biomechanical analysis capturing essential functional dynamics. The objective is to create a robust, interpretable, and potentially more data-efficient classification system capable of improving diagnostic accuracy, facilitating early risk stratification, and ultimately aiding clinical decision-making in patients with complex non-ischemic cardiomyopathies [9]. By bridging cardiovascular biomechanics and AI, this research seeks to provide a more holistic and physiologically grounded data-driven solution for the challenging diagnosis of these diverse myocardial diseases.

The core contributions of this study are as follows:

Propose a hybrid deep learning framework integrating anatomical and biomechanical features: A new type of dual-modal fusion architecture was designed, which combined the anatomical structural characteristics of CMR images with the biomechanical dynamic characteristics of IVPG time series, breaking through the limitation of traditional models that only rely on static morphological features and providing more comprehensive pathophysiological information for the classification of NICM subtypes.

Construct a dual-path network with spatiotemporal feature collaboration: Encode the spatiotemporal dynamic anatomical features of the CMR sequence through CNN-LSTM, and combine MLP to extract the pressure gradient temporal features of IVPG, achieving a deep integration of cardiac structure and functional features, and significantly improving the model’s ability to capture early subtle functional abnormalities.

The key role of verifying biomechanical characteristics through visualization tools: By using techniques such as gradient saliency and Grad-CAM, the focus of the model on key regions of the left ventricle (such as the basal septum and lateral wall) and physiological stages (diastolic rebound and early systolic) was visually demonstrated, proving the unique value of IVPG in differentiating easily confused subtypes such as HCM and CA. It provides interpretable support for model decision-making.

## 2. Materials and Methods

### 2.1. Dataset and Cohorts

This study utilized a comprehensive multi-center cohort of CMR imaging data spanning both training and validation cohorts. Standardized imaging protocols and clinical labeling criteria were implemented across all participating institutions. Data acquisition was conducted at five independent clinical centers in China under ethically approved institutional review board protocols.

#### 2.1.1. Training Set (n = 1196)

The training dataset was retrospectively collected from four large tertiary medical institutions in China, covering a diverse range of patient demographics and clinical conditions. These institutions included the following:

- Chinese PLA General Hospital (Beijing);
- Xiangya Hospital, Central South University (Changsha);
- West China Hospital, Sichuan University (Chengdu);
- Beijing Anzhen Hospital, Capital Medical University (Beijing).

Patients included in the training set completed routine cardiac assessment or clinical evaluation for alleged cardiomyopathy, undergoing comprehensive evaluations using cine CMR, echocardiography, electrocardiography, and clinical laboratory assessments. Board-certified cardiologists confirmed NICM diagnoses according to international guidelines.

Inclusion criteria required: a confirmed diagnosis; complete multi-view, multi-frame cine CMR sequences acquired using the bSSFP methodology in standard views; IVPG computation feasibility through clear endocardial tracking; and the absence of confounding conditions (e.g., ischemic heart disease, congenital defects). Corresponding echocardiographic and clinical laboratory data were also necessary for inclusion. For all cases included, manual segmentation and IVPG were computed. These cases were then carefully annotated and underwent manual quality control for imaging consistency, segment labeling, and clinical correctness, and the dataset was harmonized across sites using standardized imaging protocols and post-processing pipelines. The distribution of diagnostic classes was as follows:

- Dilated Cardiomyopathy (DCM): 474 cases;
- Hypertrophic Cardiomyopathy (HCM): 358 cases;
- Cardiac Amyloidosis (CA): 132 cases;
- Hypertensive Cardiomyopathy (HTCM): 106 cases;
- Healthy Controls (HC): 126 individuals.

#### 2.1.2. External Validation Set (n = 137)

An independent validation dataset was collected from Cangzhou People’s Hospital between 2019 and 2025. This cohort was designed to test the model’s generalizability under temporal and institutional shifts in data acquisition. Imaging and diagnosis protocols followed the same standard operating procedures used in the training centers.

The external dataset was used exclusively for model evaluation and was never involved in training or hyperparameter tuning. It represents a temporally diverse population with variations in scanner models, patient ethnicities, and subtle protocol modifications, making it ideal for robustness validation. The external set comprised the following diagnostic groups:

- Dilated Cardiomyopathy (DCM): 45 cases;
- Hypertrophic Cardiomyopathy (HCM): 35 cases;
- Cardiac Amyloidosis (CA): 22 cases;
- Hypertensive Cardiomyopathy (HTCM): 15 cases;
- Healthy Controls (HC): 20 individuals.

### 2.2. CMR Acquisition and Analysis

#### 2.2.1. CMR Image Collection

The CMR imaging was acquired using a 1.5T scanner (Philips Medical Systems, Best, The Netherlands). Cine imaging was performed in standard long-axis views (2-, 3-, and 4-chamber orientations) and short-axis stacks in all participants to extract the entire dynamic of the left ventricle. End-expiratory breath holds were performed to synchronize imaging with ECG gating and reduce motion artifacts.

With the following parameters: TR = 3.0–3.5 ms, TE = 1.5–1.8 ms, flip angle = 60°, in-plane spatial resolution = 1.5 × 1.5 mm, slice thickness = 8 mm, and temporal resolution <50 ms, the cine sequences of the bSSFP were acquired. The cine sequences had 30 frames per cardiac cycle.

#### 2.2.2. IVPG Extraction from Cine CMR

Contours and derived parameters from the myocardium, basal and mid endocardial walls, and epicardial boundaries were post-processed in Medis Suite MR (Medis Medical Imaging, version 4.1, Leiden, The Netherlands) and using QStrain (Medis Medical Imaging, version 8.1, Leiden, The Netherlands). The IVPG waveform was computed indirectly from cine CMR using a motion tracking-based approach:

- Feature-tracked endocardial borders and valve annuli were used to derive myocardial velocities.
- Spline-based interpolation generated a smooth 3D left ventricular (LV) geometry per frame.
- Inflow/outflow velocities across the mitral and aortic valve planes were also tracked.

**Figure 1:**
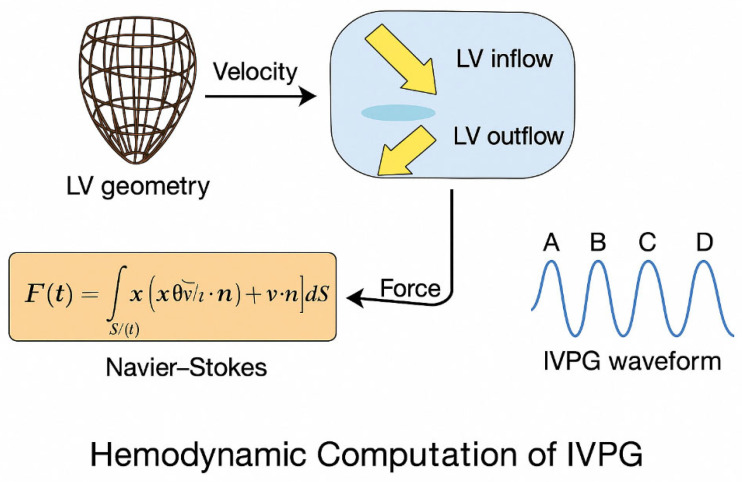
Hemodynamic computation pipeline for IVPG estimation from cine CMR data.

This approach was used to compute IVPGs as biomechanical markers of cardiac function. The feature-tracked motion of endocardial surfaces, mitral and aortic valve annuli, and their intersection with a sheared spline were used to reconstruct a 3D LV geometry for each frame. These velocity fields were input into a force-based model to estimate the pressure gradient (Figure 1).

(1) Volumetric Force Derivation

The net intraventricular force was estimated using the unsteady Navier–Stokes momentum equation:

$$\;F\left(t\right)=\rho {∭}_{V\left(t\right)}\left(-\frac{\partial v}{\partial t}-\left(v\cdot \nabla \right)v\right)dV$$

where ρ is blood density (1.0 kg/L), v is the velocity vector, and $V\left(t\right)$ is the LV cavity volume.

(2) Surface Reformulation via Gauss Theorem

To simplify numerical implementation, Gauss’ divergence theorem was applied:

$$\;F\left(t\right)=\rho \iint_{S\left(t\right)}\left[x\left(\frac{\partial v}{\partial t}\cdot n\right)+v\left(v\cdot n\right)\right]dS$$

where x is the position vector, $S\left(t\right)$ the LV surface, and n the outward normal vector.

(3) Segmentation into Functional Phases

The resulting IVPG waveform, aligned with CMR frame timing, was segmented into four key physiological intervals:

- A-wave: Systolic ejection;
- B-wave: Early diastolic suction (recoil);
- C-wave: Passive filling;
- D-wave: Atrial contraction.

Each segment encapsulates diagnostic biomechanical cues. IVPG sequences were downsampled and normalized before being input into the MLP encoder.

### 2.3. Artificial Intelligence Methodology

**Figure 2:**
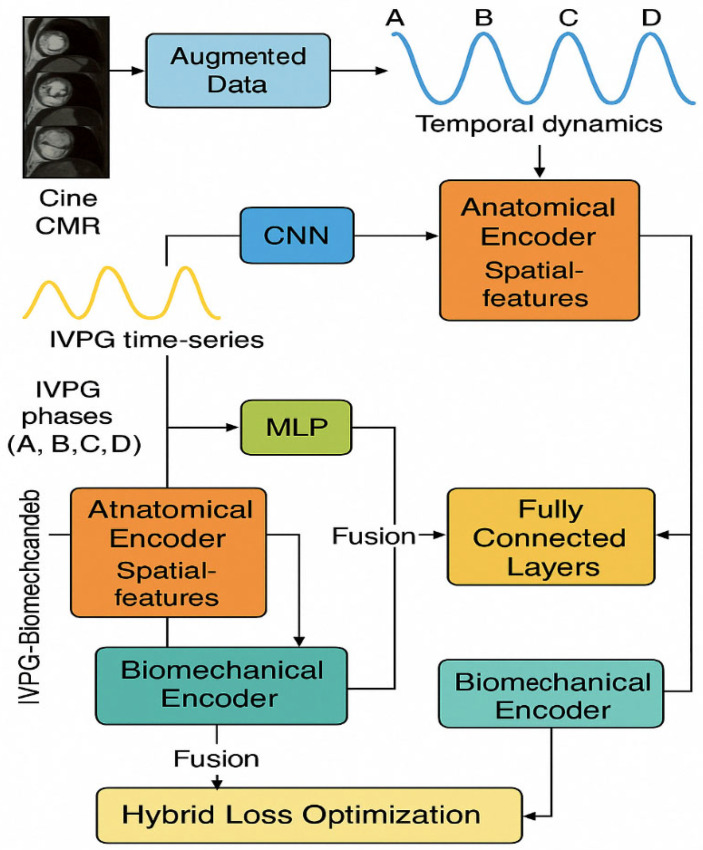
Hybrid deep learning model combining CMR and IVPG features for NICM classification. This diagram represents a hybrid deep learning model designed to classify NICM by combining anatomical and biomechanical data. Cine CMR images are processed through a CNN and LSTM to extract spatiotemporal features, while IVPG time-series signals are encoded using an MLP to capture cardiac pressure dynamics. The outputs from both streams are fused and passed through dense layers and a softmax classifier for final prediction. The model also uses GAN-based augmentation to increase CMR data diversity and a hybrid loss function to improve training stability and physiological relevance.

We used the complete fusion system, as shown in Figure 2 (integrating the anatomy of CMR and the biomechanical characteristics of IVPG), to classify NICM. The system enhances data diversity through generative adversarial networks (GANs) and designs dual-path encoders to achieve multimodal feature collaboration. This is a system combining two types of data: CMR images and the IVPG time-series data. First, a technique called generative adversarial network (GAN)-based data augmentation is used to enhance the images before them so that the model is more accurate and reliable. Second, these images are then taken and fed into a CNN (convolutional neural network) to identify the important features. Moreover, the IVPG data goes through another model, i.e., the multi-layer perception (MLP), intended to extract useful information from pressure signals. After this, the features extracted from both types of data are subjected to fully connected layers, to allow the model to become better. Finally, the prediction of the type of NICM is made by the system using a softmax classifier.

The development of and training of a hybrid dual-stream architecture are based on anatomical (CMR) and biomechanical features (IVPG) with a holistic concept, transfer learning, feature alignment, and biomechanics sensitivity regularization for improved interpretation and generalization. We would use the proposed model to internally and externally validate it via metrics such as accuracy, F1-score, AUC, and gradient-based interpretability analyses against baseline AI methods (e.g., ResNet50, VGG16, SVM with radiomics).

#### 2.3.1. Mathematical Formulation

Let $D={\left\{\left(X_{i},V_{i},y_{i}\right)\right\}}_{i=1}^{N}$ be a dataset of N samples, where $X_{i}\in {\mathbb{R}}^{T\times H\times W\times C}$ is the temporal cine-CMR sequence, $V_{i}\in {\mathbb{R}}^{T\times 3}$ is the corresponding biomechanical IVPG vector sequence, and $y_{i}\in \left\{1,2,\ldots ,K\right\}$ is the ground-truth NICM class label.

The model $F_{\theta }\left(X_{i},V_{i}\right)$ parameterized by weights θ is trained by minimizing the following loss:

(1) $$\;\underset{\theta }{\min}\;L_{\mathrm{CE}}\left(F_{\theta }\left(X_{i},V_{i}\right),y_{i}\right)+\lambda \|\nabla_{V}F_{\theta }{\|}^{2}+\mu \cdot \Omega \left(\theta \right)$$

where $L_{\mathrm{CE}}$ is the categorical cross-entropy loss, $\|\nabla_{V}F_{\theta }{\|}^{2}$ is a biomechanical sensitivity regularizer, $\Omega \left(\theta \right)$ is a standard regularization term (e.g., $l_{2}$ norm), and λ, μ are scalar hyperparameters.

#### 2.3.2. Objective Functions

(1) Feature alignment objective:

(2) $$\;\underset{\theta }{\min}\;E_{i∼D}\left[D_{\mathrm{KL}}\left(f_{X}\left(X_{i}\right)∥f_{V}\left(V_{i}\right)\right)\right]$$

(2) Classification objective:

(3) $$\;\underset{\theta }{\min}\;\overset{N}{\underset{i=1}{\sum }}L_{\mathrm{CE}}\left(F_{\theta }\left(X_{i},V_{i}\right),y_{i}\right)$$

(3) Biomechanical interpretability objective:

(4) $$\;\underset{\theta }{\min}\;E\left[\|\nabla_{V_{i}}F_{\theta }{\left(X_{i},V_{i}\right)\|}^{2}\right]$$

Notations used:

– $X_{i}$: Multi-frame CMR sequence for subject i;
– $V_{i}$: Time-series IVPG vectors for subject i;
– $y_{i}$: True diagnostic label (NICM subtype);
– T,H,W,C: Temporal length, height, width, and channel count of CMR data;
– $F_{\theta }$: Deep classification model with learnable parameters θ;
– $\nabla_{V}$: Gradient operator biomechanical input V;
– λ,μ: Regularization coefficients;
– $D_{\mathrm{KL}}$: Kullback–Leibler divergence;
– $\Omega \left(\theta \right)$: Weight regularization function.

#### 2.3.3. Dual-Stream AI Architecture

(1) Anatomical Encoding via CNN-LSTM

CMR sequences ($X_{i}\in {\mathbb{R}}^{T\times H\times W\times C}$) were processed as follows:

- CNN (ResNet-50): Extracts spatial features frame-wise from $X_{t}$.
- LSTM: Two-layer LSTM encodes cardiac motion dynamics over time:

$$\;h_{t}=\mathrm{LSTM}\left(f_{\mathrm{CNN}}\left(X_{t}\right),h_{t-1}\right)$$

where $h_{t}$ is the hidden state and $f_{\mathrm{CNN}}\left(X_{t}\right)$ the CNN-extracted feature.

The final LSTM state is used as the anatomical embedding.

(2) Biomechanical Encoding via MLP

Time-aligned IVPG vectors ($V_{i}\in {\mathbb{R}}^{T\times 3}$) were input into a 3-layer MLP:

- Dense (64) → ReLU;
- Dense (32) → ReLU;
- Dense (16) → Embedding output.

(3) Fusion and Final Classification

The outputs from both streams were concatenated and passed through:

- Dense (128) → ReLU;
- Dense (64) → ReLU;
- Dropout (0.4);
- Softmax output for 5-class NICM prediction.

(4) GAN-Based Augmentation Strategy

To mitigate overfitting and enhance variability, a GAN was trained on CMR sequences with the following objective:

$$\;\underset{G}{\min}\underset{D}{\max}E_{x∼p_{\mathrm{data}}}\left[\log D\left(x\right)\right]+E_{z∼p_{z}}\left[\log\left(1-D\left(G\left(z\right)\right)\right)\right]$$

After visual screening by cardiologists, the generated samples are used exclusively for data augmentation within the training set. To prevent data leakage, these samples are excluded from both validation and test sets during model evaluation. The model training uses the NVIDIA Tesla V100 GPU. Each epoch takes approximately 15 min, and a total of 100 epochs are trained. The total number of parameters is as follows: approximately 2.3 million CNN-LSTM paths, approximately 12,000 MLP paths, approximately 150,000 fusion layers, totaling approximately 2.462 million parameters.

(5) Model Training Strategy

The training/validation set is divided by using 5-fold cross-validation. The external validation set is independent of the training process and does not participate in any parameter adjustment. The model was optimized using a multi-objective loss:

$$\;L=L_{\mathrm{CE}}+\lambda ∥\nabla_{V}F_{\theta }{∥}^{2}+\mu \cdot \Omega \left(\theta \right)$$

- $L_{\mathrm{CE}}$: Categorical cross-entropy;
- $∥\nabla_{V}F_{\theta }{∥}^{2}$: IVPG sensitivity regularization;
- $\Omega \left(\theta \right)$: L2 weight decay.

(6) Hyperparameters

Hyperparameters were optimized using a grid search over the validation set to balance classification accuracy and biomechanical sensitivity. The final values—Adam optimizer with learning rate $1\times 10^{-4}$, batch size = 16, epochs = 100, λ=0.1, and μ=0.001—were selected to maximize the validation AUC. Specifically, λ (biomechanical sensitivity regularizer) and μ (L2 weight decay) were tuned across ranges 0.01≤λ≤0.5 and 0.001≤μ≤0.01, with the chosen values demonstrating the best trade-off between preventing overfitting and ensuring the model remained sensitive to IVPG dynamics.

Model training was conducted using a 5-fold cross-validation strategy. The training data were partitioned into five subsets, with each iteration using four subsets for training and one for validation. The final internal validation results represent the average performance across all five folds. An independent external validation set, which was not involved in any stage of training or hyperparameter tuning, was used exclusively to evaluate the final model performance. The generated samples were evaluated by cardiologists to ensure the authenticity of the left ventricular anatomy was preserved. To verify the effectiveness of GAN enhancement, we compared the model performance with and without GAN data: In the 5-fold cross-validation, the internal AUC increased from 0.961 to 0.974 after adding GAN data, indicating that reasonable synthetic data can alleviate overfitting without introducing significant errors.

### 2.4. Ethical Compliance and Data Handling

The data were de-identified according to the institutional review board and the Declaration of Helsinki. Each participating center obtained ethical approval for secondary research use. It was also without the retention of any personally identifiable information. We stored data on secure, encrypted servers that were available for only approved personnel. Each consensually confirmed clinical label was marked on all participants after obtaining informed consent.

## 3. Results

### 3.1. Quantitative Performance Evaluation

The internal validation results are based on the average performance across a 5-fold cross-validation (n = 1196), offering a robust evaluation while minimizing overfitting. Compared to ResNet50, which contains 25.5 million parameters, the proposed model reduces the parameter count by 89% through a dual-path lightweight architecture, while still achieving a superior classification performance, with an external AUC improvement of +0.044. This highlights the model’s strong data efficiency.

To enhance diagnostic accuracy, the hybrid AI model integrates biomechanical features extracted from cine CMR with IVPG-derived physiological indicators. As shown in Table 1, the model achieved an internal cross-validation accuracy of 94.2%, with a precision of 93.8%, recall of 94.2%, F1-score of 93.4%, and AUC of 0.974, indicating an excellent performance and consistency across centers. The external validation results (n = 137) yielded an accuracy of 92.1%, with a similarly high performance in all metrics, further confirming the model’s generalizability and clinical applicability.

**Figure 3:**
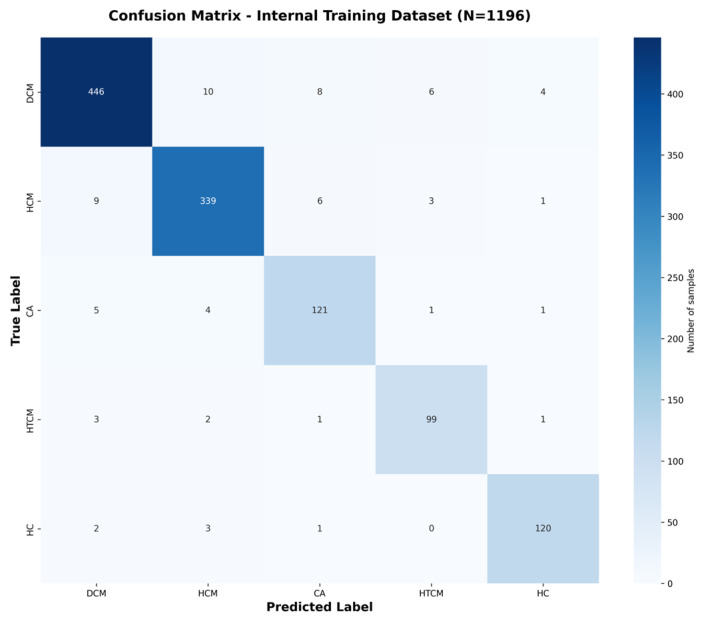
Confusion matrix for internal validation dataset classification. Confusion matrix for the internal dataset (N = 1196), obtained by pooling predictions across all five folds of cross-validation.

**Figure 4:**
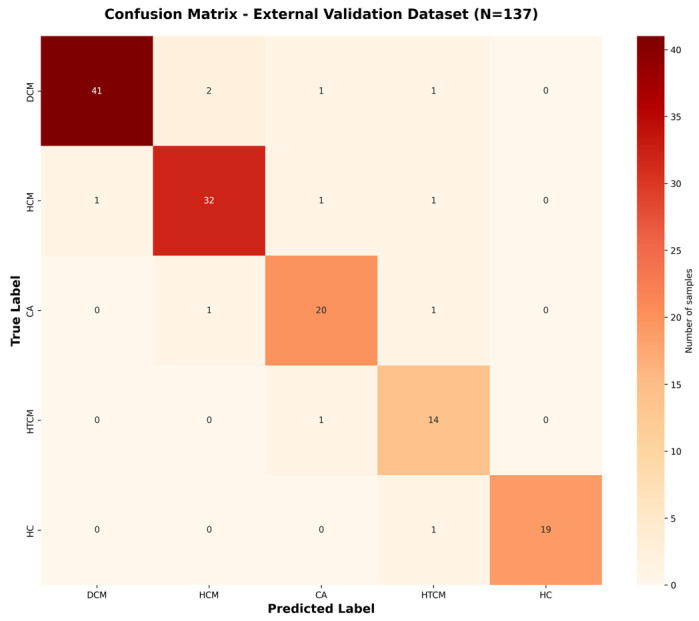
Confusion matrix for external dataset classification. Confusion matrix for the external dataset (N = 137), based on predictions from the trained final model.

Confusion matrices (Figure 3 and Figure 4) are shown, which illustrate the high sensitivity in classifying DCM and HCM alone, with a slight confusion between CA and HTCM.

All classification metrics, including accuracy, precision, recall, and F1-score, were computed using a weighted average approach to account for class imbalance across categories. Cohen’s Kappa coefficient was calculated using the scikit-learn library, based on the following formula:

$$\;\kappa =\frac{agreement-expected\;agreement}{1-expected\;agreement}$$

Here, agreement denotes the observed classification consistency, while expected agreement represents the agreement expected by random chance.

It then evaluated the biomechanical integration added value compared with baseline models. The results indicated that in external validation, the hybrid model outperformed the baseline model in terms of accuracy, F1-score, AUC and Kappa performance indicators (Table 2).

To evaluate the statistical significance of performance differences among models, DeLong’s test was employed for pairwise comparisons of the area under the ROC curve (AUC) values [9]. The proposed hybrid model (CMR + IVPG), which achieved an AUC of 0.962, demonstrated a statistically significant improvement over the ResNet50 model (CMR only, AUC = 0.918; *p* < 0.001). Furthermore, the hybrid model also outperformed the VGG16 (AUC = 0.909) and Radiomics + SVM (AUC = 0.892) models with statistical significance (*p* < 0.01 for both). These results provide robust evidence that the enhanced classification performance of the proposed model—attributable to the integration of biomechanical features—is not incidental, but is underpinned by a solid statistical foundation.

**Figure 5:**
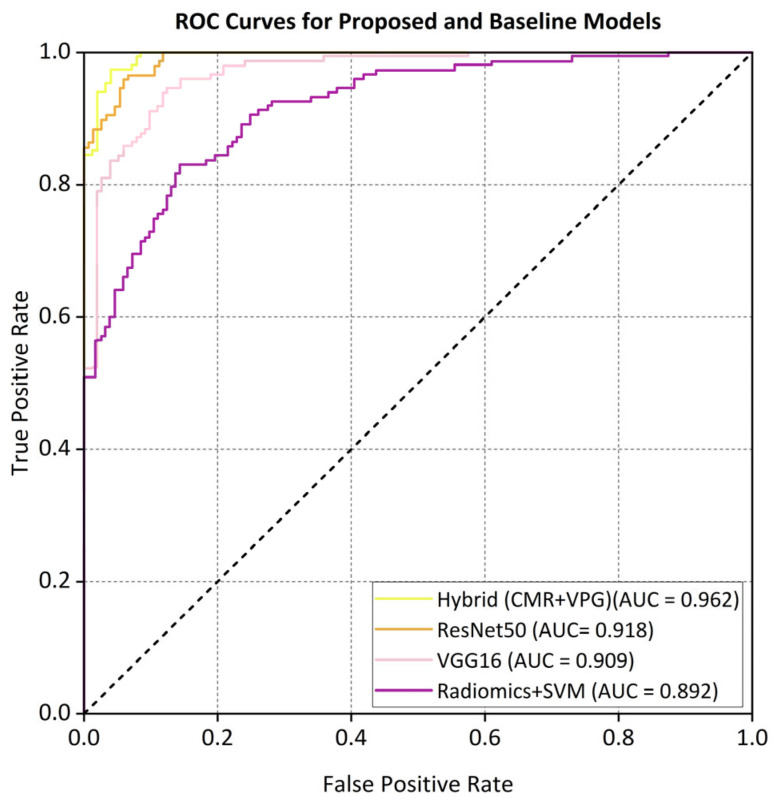
ROC curves comparing different models on the external dataset.

**Figure 6:**
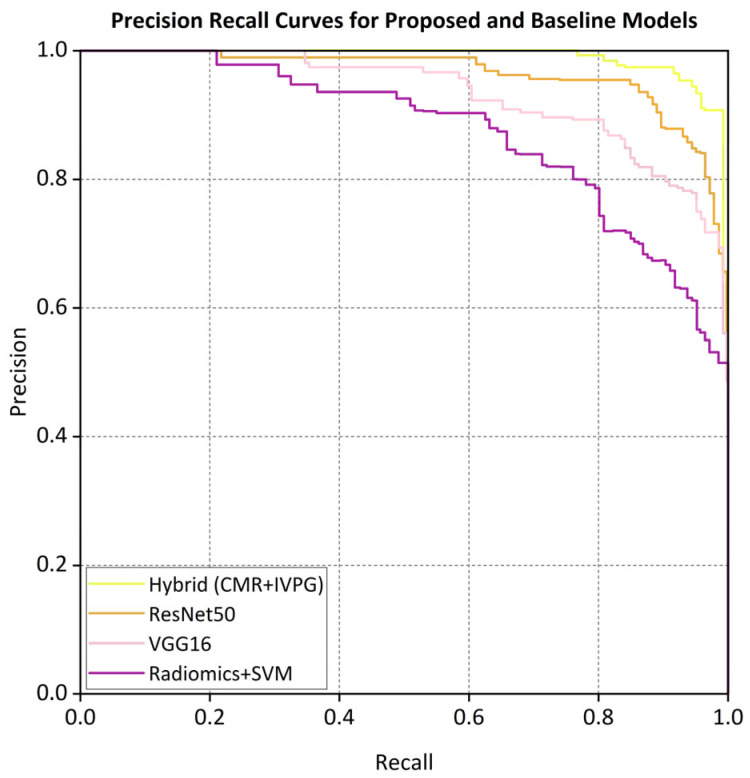
Precision-recall curves for proposed and baseline models.

The ROC curve (Figure 5) and the precision–recall (PR) curves (Figure 6) demonstrate that the proposed hybrid model consistently outperforms the baseline models in the classification of non-ischemic cardiomyopathy (NICM).

The proposed hybrid model achieved strong discriminative performance on the external validation set, with a micro-average AUC of 0.962. As illustrated in Figure 5, the AUC values for each class in the one-vs.-rest setting were a DCM (0.958), HCM (0.965), CA (0.942), HTCM (0.931), and HC (0.970). In addition, the precision–recall (PR) curve shown in Figure 6 indicates that the model attained a precision of 91% at a recall of 80%, clearly outperforming all baseline models in predictive accuracy.

### 3.2. Contribution of IVPG Features

An ablation study of IVPG input was also performed. The results are shown in Table 3.

**Figure 7:**
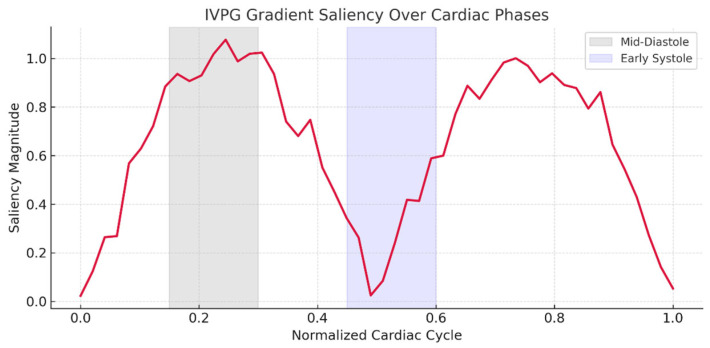
IVPG gradient saliency highlighting biomechanical sensitivity zones.

Gradient saliency maps (Figure 7) demonstrated the importance of IVPG patterns near left ventricular recoil (+the physiologically corresponding left ventricular peak filling pressure) and near early systole (+early systole physiologically correlated with the peak filling pressure).

**Figure 8:**
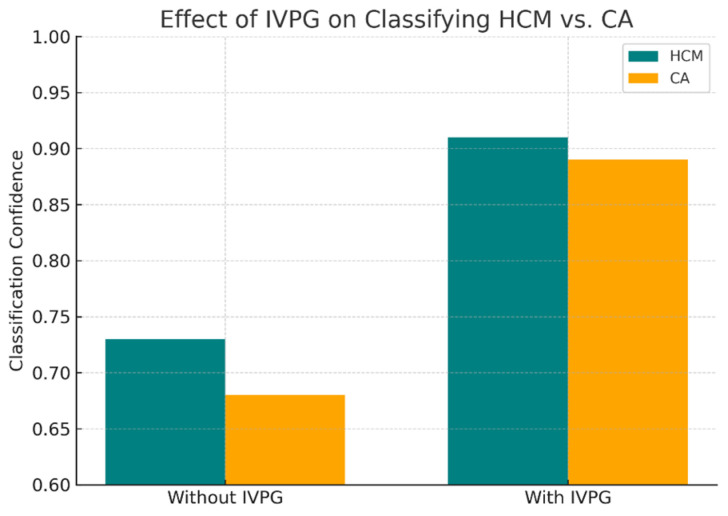
Improved classification of HCM vs. CA using biomechanical cues.

IVPG provides enhanced discriminative power in borderline cases, such as to distinguish cardiac amyloidosis from hypertrophic cardiomyopathy (Figure 8).

### 3.3. Generalizability and External Validation

The model was shown to perform with minimal degradation to its performance when deployed at an external site (Cangzhou People’s Hospital), and hence is generalizable across institutions (Table 4).

It was shown that temporal robustness does not suffer from significant accuracy fluctuations over changing imaging protocols.

### 3.4. Model Interpretability and Clinical Relevance

**Figure 9:**
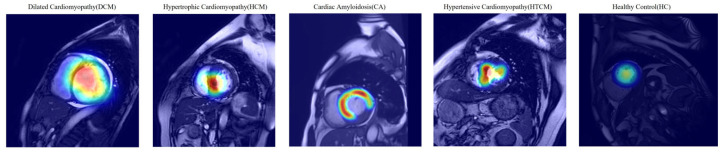
Grad-CAM visualizations of model attention on cine CMR images for different cardiomyopathy subtypes. The heat maps show regions of high model attention (red/yellow regions) overlaid on CMR images. For dilated cardiomyopathy (DCM), the model focuses on the enlarged cardiac cavity edges and left ventricular wall dilation. In hypertrophic cardiomyopathy (HCM), attention is primarily concentrated on the thickened interventricular septum at the ventricular base. For cardiac amyloidosis (CA), the model demonstrates diffuse attention patterns across the myocardium, reflecting the characteristic diffuse myocardial enhancement. The visualizations confirm that the model learns clinically relevant anatomical features, with attention directed to pathognomonic regions specific to each cardiomyopathy subtype.

The Grad-CAM visualizations illustrate distinct model attention patterns across the five cardiomyopathy subtypes, as shown in Figure 9. For dilated cardiomyopathy (DCM), the model focuses on the enlarged left ventricular cavity and lateral wall, reflecting chamber dilation. In hypertrophic cardiomyopathy (HCM), attention is concentrated on the thickened interventricular septum. Cardiac amyloidosis (CA) exhibits diffuse attention across the entire myocardium, consistent with global myocardial thickening. In hypertensive cardiomyopathy (HTCM), the model highlights localized thickening along the septum and basal lateral wall. For healthy controls (HCs), attention is minimal and diffusely distributed, indicating a lack of pathological features.

These results suggest that the model learns to identify disease-specific anatomical patterns, with attention directed to clinically relevant regions for each subtype. The white outlines in the heatmaps indicate key diagnostic zones that potentially inform model-based decision-making.

**Figure 10:**
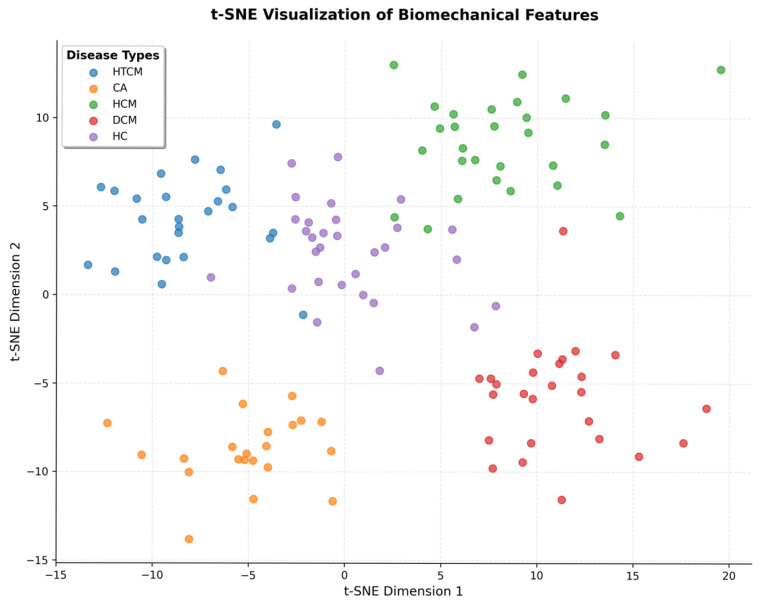
t-SNE plot of learned biomechanical feature embeddings by class.

These embeddings were then projected onto the t-SNE (Figure 10), where biomes bestowed by different diseases had distinct clusters.

The interpretability components in anatomical and physiological space both serve to increase the model’s potential clinical adoption where the anatomical presentation is ambiguous.

It should be noted that these visualization results reflect characteristic correlations rather than causal relationships. The specific pathological mechanisms need to be further verified in combination with clinical and basic research.

### 3.5. Comparative Analysis of Model Variants

**Figure 11:**
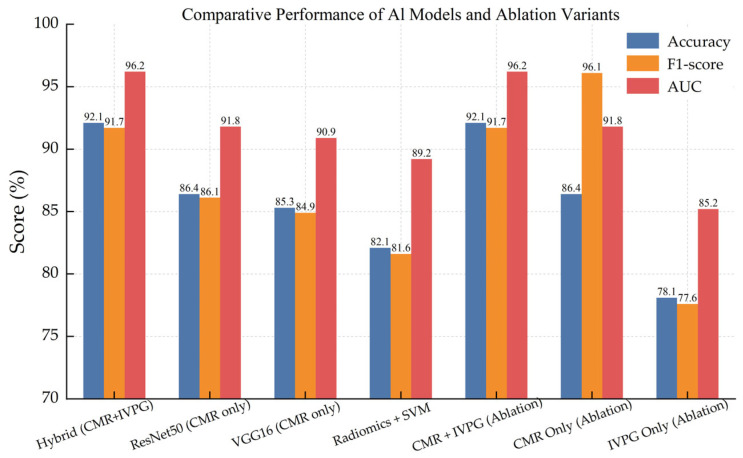
Comparative bar chart showing accuracy, F1-score, and AUC across all model variants, including baseline CNNs, radiomics-based classifiers, and ablation studies.

We comprehensively compare across the main configurations in order to understand in detail the contribution of biomechanical features and the benchmark against baseline architectures with the proposed hybrid model. Finally, Table 5 and Figure 11 show a summary of the performance of each model variant in terms of accuracy, F1-score, AUC, and Cohen’s Kappa coefficient.

It is shown undoubtedly that the given model has outperformed all the baseline models on all metrics. Interestingly, the IVPG-only model still showed moderate discriminative power on behalf of the physiological relevance of biomechanical features.

This not only confirms the good performance of integrating anatomical and biomechanical signals for dependable and interpretable NICM classification but also supports the design of the AI framework specified.

## 4. Discussion

The application of deep learning (DL) to automate the classification of NICMs in CMR imaging has emerged as a transformative paradigm in cardiovascular diagnostics. By leveraging hierarchical feature extraction and robust image interpretation, DL models have consistently outperformed traditional radiomic approaches in distinguishing NICM subtypes. For instance, Nakashima et al. [10] pioneered the use of CNNs to differentiate ischemic cardiomyopathy from NICM using CMR cine imaging, achieving 92% accuracy—a significant improvement over handcrafted radiomic features. Subsequent work by the same group [11] demonstrated the value of anatomically constrained learning, where segmentation-guided CNNs improved classification AUC from 0.87 to 0.94. These findings underscore the synergistic potential of integrating anatomical priors with DL architectures to enhance diagnostic precision.

Notably, while classical machine learning methods such as k-nearest neighbors and support vector machines (SVMs) have achieved competitive accuracy (91.7%) in NICM classification [12], their reliance on static radiomic features limits their ability to capture dynamic pathophysiological processes. For example, Jiang et al. [13] reported an AUC of 0.95 in distinguishing CA from HCM using non-contrast cine MRI radiomics, yet their workflow required computationally intensive preprocessing, which hinders clinical adoption. Similarly, Avard et al. [14] emphasized the diagnostic utility of radiomics in detecting myocardial infarction on non-contrast cine MRI but overlooked the integration of biomechanical modeling, a critical gap given the hemodynamic complexity of NICMs. Collectively, these studies highlight a persistent challenge: achieving generalizable, interpretable, and physiologically grounded classification requires moving beyond conventional imaging features to incorporate functional and biomechanical biomarkers.

Recent efforts to address these limitations have focused on patient-specific, contrast-free approaches that enhance clinical applicability. Miller et al. [15] developed a CNN-based pipeline for simulating patient-specific biventricular mechanics, revealing enhanced biomechanical insights into myocardial motion. However, the computational intensity of their method precludes real-world clinical integration. In parallel, Abdulkareem et al. [16] demonstrated the feasibility of predicting post-contrast MRI information from non-contrast sequences using regression-based machine learning, achieving <5% mean absolute error—though performance variability across patient cohorts underscored the need for harmonized training data. These advancements align with recommendations by Anand and Janardhanan [17], who advocate for augmenting CMR’s diagnostic utility through quantitative biomechanical biomarkers rather than relying solely on volumetric assessments. Wang et al. [18] further emphasized the critical role of DL in NICM subtyping when anatomical and tissue characterization features are ambiguous, while Deng et al. [19] identified translational barriers such as data heterogeneity, interpretability deficits in “black-box” DL systems, and the lack of standardized pipelines. Together, these works lay the groundwork for hybrid models that synergize DL with pathophysiological biomarkers to improve classification accuracy and clinical trust.

Cardiac biomechanics have emerged as a pivotal complement to conventional imaging in unraveling NICM pathophysiology. IVPGs, in particular, have gained recognition as sensitive markers of systolic and diastolic dysfunction. El-Husseiny et al. [20] first established IVPG’s diagnostic utility in chronic congestive heart failure, where it detected residual myocardial dysfunction missed by standard volumetric metrics. Subsequent studies by Konijnenberg et al. [21] linked persistent IVPG abnormalities to adverse remodeling in ST-segment elevation myocardial infarction, while Hirose et al. [22] demonstrated the clinical relevance of mitral inflow dynamics in hypertensive cardiomyopathy. Tang et al. [23] further correlated CMR-derived myocardial microstructural changes with altered IVPG waveforms in NICMs, and Jumadilova et al. [24] revealed IVPG’s sensitivity to both physiological (athletic adaptation) and pathological (hypertensive) remodeling. Despite these advances, IVPG quantification has historically been constrained by its dependence on high-resolution imaging and assumptions embedded in computational fluid dynamics models—limitations now being overcome through AI-driven innovations.

The integration of physics-informed neural networks (PINNs) and biomechanical modeling represents a paradigm shift in non-invasive IVPG estimation. Wong et al. [25] pioneered this approach, reconstructing 3D velocity and pressure fields from echocardiography to estimate IVPGs without invasive measurements, albeit with trade-offs in model complexity and Doppler data dependency. Meyers [26] later demonstrated IVPG extraction feasibility from routine echocardiographic workflows, though real-time accuracy remains suboptimal. Translational studies by Fatehi Hassanabad and Garcia [27] linked ventricular kinetic energy and IVPGs to surgical outcomes in bicuspid aortic valve patients, while Sugiura et al. [28] proposed IVPG-guided regenerative strategies for heart failure. At the molecular level, Hu et al. [29] integrated IVPG-based biomechanical signatures with multi-omics data to trace cardiomyopathy pathogenesis—a systems biology approach with profound implications for personalized therapy. These innovations collectively underscore IVPG’s dual role as a diagnostic biomarker and a dynamic modulator of cardiac remodeling, positioning it as an indispensable component of next-generation AI models.

Our hybrid AI model, which synergizes CMR-derived anatomical features with IVPG-based biomechanical biomarkers, achieved a state-of-the-art classification performance across internal (n = 1196) and external (n = 137) validation cohorts. On internal testing, the model attained 94.2% accuracy, a 93.4% F1-score, and 0.974 AUC—metrics that remained robust in external validation (92.1% accuracy, 91.7% F1, 0.962 AUC) despite variations in MRI protocols over a five-year span (Table 4). Comparative analysis against established baselines revealed substantial improvements: the hybrid model outperformed ResNet50 (86.4%), VGG16 (85.3%), and radiomics-based SVM (82.1%) by 6–12 percentage points in external accuracy (Table 2). ROC and precision–recall analyses (Figure 5 and Figure 6) confirmed the superior discriminative capacity across all NICM subtypes, particularly HCM and CA, where classification confidence improved from 73% to 91% and 68% to 89%, respectively (Figure 8).

Ablation studies highlighted IVPG’s critical contribution: excluding biomechanical features reduced external accuracy by 6.3% (Table 3), though standalone IVPG analysis retained a modest diagnostic value (AUC = 0.852). This suggests that while IVPGs alone capture limited pathophysiology, their synergy with structural imaging enables the model to learn physiologically meaningful representations—a hypothesis corroborated by saliency maps (Figure 7) and Grad-CAM visualizations (Figure 9). The former revealed heightened model attention to IVPG dynamics during mid-to-late diastole, coinciding with left ventricular recoil and rapid filling phases, while the latter localized discriminative features to clinically relevant myocardial regions (basal septum and lateral wall). Furthermore, t-SNE projections (Figure 10) demonstrated distinct clustering of NICM subtypes in biomechanical embedding space, confirming the model’s ability to disentangle complex pathophysiological signatures.

This study proposes a novel hybrid AI model that integrates CMR imaging features with IVPG biomechanical biomarkers, advancing beyond traditional frameworks reliant on static anatomical or textural features. Compared to existing deep learning models, our hybrid model demonstrates a significantly superior classification performance in both internal and external validations (external accuracy: 92.1% vs. baseline models at 86.4%). Notably, it is the first to incorporate time-resolved IVPG dynamics, capturing pathophysiological mechanisms during mid-to-late diastolic phases (e.g., left ventricular recoil and rapid filling), thereby addressing a critical gap in prior studies that overlooked dynamic biomechanical signatures. Furthermore, the model achieves anatomical and functional interpretability through Grad-CAM and t-SNE visualizations, pinpointing clinically relevant myocardial regions (e.g., basal septum and lateral wall) that drive classification decisions. This transparency bridges the “black-box” dilemma of AI, offering a new paradigm for explainable diagnostics. The model’s robustness across diverse clinical settings (AUC 0.961–0.964 under varying MRI protocols over five years) underscores its potential for real-world deployment, laying a foundation for precision subtyping of NICMs based on multimodal biomechanical profiling.

Despite these advancements, several limitations must be acknowledged. First, while the study utilized multicenter data, all training and validation cohorts were derived from Chinese healthcare institutions. Genetic heterogeneity, regional variations in disease etiology, and differences in clinical practices may constrain the model’s generalizability to global populations. Second, IVPG quantification requires high-spatiotemporal-resolution CMR cine sequences, which may not be feasible in resource-limited settings using low-field scanners or rapid imaging protocols. Third, although saliency maps and Grad-CAM visualizations enhance interpretability, they reveal feature correlations rather than causal relationships. For instance, whether IVPG abnormalities directly drive NICM phenotypes or merely reflect secondary hemodynamic adaptations remains unclear. Future studies integrating in vitro flow simulations or genotype–phenotype analyses are needed to validate these biomechanical–pathophysiological linkages.

Although this study has achieved promising results, there are still several limitations. Firstly, all training and validation data are from Chinese medical institutions. The genetic background, disease etiology, and clinical management strategies of the patient population may have regional specificity, which may affect the generalization ability of the model to different races and medical environments around the world. Secondly, the accurate calculation of IVPG relies on CMR cine sequences with high-spatiotemporal-resolution (such as the time resolution of bSSFP sequences < 50 ms) and professional post-processing tools (such as Medis Suite MR), which may be difficult to achieve in medical institutions with limited resources or low-field-strength MRI equipment. It limits the wide application of this technology. Furthermore, although visualization tools such as Gradient-CAM enhance the transparency of the model, such methods can only reveal the correlation between features and classification decisions, rather than the causal relationship. For example, the signal changes in the basal segment of the ventricular septum that the model focuses on may be related to NICM subtypes, but it cannot be directly proven as a pathogenic mechanism. Future research can further enhance the practicability and explanatory depth of the model through multi-center global cohort validation, simplifying the calculation process of IVPG, and combining basic experiments to verify the pathophysiological significance of biomechanical characteristics. The high requirements of IVPG calculation for CMR equipment and post-processing procedures may limit its application in primary hospitals or institutions using old MRI models. Future research can explore simplified IVPG estimation methods based on low-resolution sequences, or develop lightweight AI tools to lower the computational threshold.

## 5. Conclusions

By harmonizing deep learning with biomechanical biomarkers, this study advances NICM classification beyond the limitations of conventional imaging-based models. The hybrid architecture not only achieves a superior diagnostic accuracy but also provides interpretable insights into myocardial dysfunction mechanisms—a critical step toward personalized cardiomyopathy management. As AI continues to reshape cardiovascular diagnostics, our work underscores the transformative potential of unifying data-driven learning with pathophysiological principles to bridge the gap between computational innovation and clinical translation.

## Figures and Tables

**Table 1:** Classification performance metrics on internal and external datasets.

Dataset	Accuracy	Precision	Recall	F1-Score	AUC	Kappa
Internal (n = 1196)	94.2%	93.8%	94.2%	93.4%	0.974	0.925
External (n = 137)	92.1%	92.3%	92.1%	91.7%	0.962	0.884

Internal validation is based on the weighted average index of 5-fold cross-validation, and external validation is calculated based on the weighted average of independent test sets.

**Table 2:** Performance comparison with baseline models (external dataset).

Model	Accuracy	F1-Score	AUC	Kappa
Proposed hybrid (CMR + IVPG)	92.1%	91.7%	0.962	0.884
ResNet50 (CMR only)	86.4%	86.1%	0.918	0.812
VGG16 (CMR only)	85.3%	84.9%	0.909	0.799
Radiomics + SVM	82.1%	81.6%	0.892	0.752

**Table 3:** Ablation study results on the external dataset.

Model Variant	Accuracy	F1-Score	AUC
CMR + IVPG (full model)	92.1%	91.7%	0.962
CMR only	86.4%	86.1%	0.918
IVPG only	78.1%	77.6%	0.852

**Table 4:** External performance by year and site.

Subset	Accuracy	F1-Score	AUC
2019–2020	92.3%	91.8%	0.961
2021–2023	91.9%	91.5%	0.963
2024–2025	92.0%	91.6%	0.964
Cangzhou (overall)	92.1%	91.7%	0.962

**Table 5:** Comparative performance of proposed and baseline models with ablation variants.

Model	Accuracy	F1-Score	AUC	Kappa
Hybrid (CMR + IVPG)	92.1%	91.7%	0.962	0.884
ResNet50 (CMR only)	86.4%	86.1%	0.918	0.812
VGG16 (CMR only)	85.3%	84.9%	0.909	0.799
Radiomics + SVM	82.1%	81.6%	0.892	0.752
CMR + IVPG (ablation test)	92.1%	91.7%	0.962	0.884
CMR only (ablation test)	86.4%	86.1%	0.918	0.812
IVPG only (ablation test)	78.1%	77.6%	0.852	–

## Data Availability

In this study, there are ethical constraints regarding the sharing of de-identified data. The ethics committee has not given approval for the public sharing of the data since we did not obtain consent from the participants to share their anonymized data. Considering the nature of the dataset, despite our efforts to anonymize the data, the possibility remains that patients could still be identifiable. The code implementation is based on the PyTorch 2.5 framework. scikit-learn 1.2.2 and TensorFlow 2.10 were used for metric calculation and visualization. Eligible and interested researchers can acquire the data by contacting the authors.

## Abbreviations

The following abbreviations are used in this manuscript:

NICMs	Non-ischemic cardiomyopathies
IVPGs	Intraventricular pressure gradients
CMR	Cardiac magnetic resonance
DCM	Dilated cardiomyopathy
HCM	Hypertrophic cardiomyopathy
HTCM	Hypertensive cardiomyopathy
CA	Cardiac amyloidosis
CNNs	Convolutional neural networks
GAN	Generative adversarial network
MLP	Multi-layer perception
DL	Deep learning
SVMs	Support vector machines
Grad-CAM	Gradient-weighted class activation mapping
